# Analytical Model for Estimating the Zenith Angle Dependence of Terrestrial Cosmic Ray Fluxes

**DOI:** 10.1371/journal.pone.0160390

**Published:** 2016-08-04

**Authors:** Tatsuhiko Sato

**Affiliations:** Research Group for Radiation Transport Analysis, Japan Atomic Energy Agency (JAEA), Shirakata 2–4, Tokai, Ibaraki 319–1195, Japan; North Shore Long Island Jewish Health System, UNITED STATES

## Abstract

A new model called “PHITS-based Analytical Radiation Model in the Atmosphere (PARMA) version 4.0” was developed to facilitate instantaneous estimation of not only omnidirectional but also angular differential energy spectra of cosmic ray fluxes anywhere in Earth’s atmosphere at nearly any given time. It consists of its previous version, PARMA3.0, for calculating the omnidirectional fluxes and several mathematical functions proposed in this study for expressing their zenith-angle dependences. The numerical values of the parameters used in these functions were fitted to reproduce the results of the extensive air shower simulation performed by Particle and Heavy Ion Transport code System (PHITS). The angular distributions of ground-level muons at large zenith angles were specially determined by introducing an optional function developed on the basis of experimental data. The accuracy of PARMA4.0 was closely verified using multiple sets of experimental data obtained under various global conditions. This extension enlarges the model’s applicability to more areas of research, including design of cosmic-ray detectors, muon radiography, soil moisture monitoring, and cosmic-ray shielding calculation. PARMA4.0 is available freely and is easy to use, as implemented in the open-access EXcel-based Program for Calculating Atmospheric Cosmic-ray Spectrum (EXPACS).

## Introduction

High-energy galactic cosmic rays (GCR) can penetrate the Earth’s magnetosphere and produce a variety of secondary particles in the atmosphere by inducing extensive air shower (EAS). Estimation of terrestrial cosmic-ray fluxes is of great importance not only for particle physics and astrophysics but also for the geosciences and radiation research. Thus, a number of studies were devoted for their evaluation on the basis of analytical approaches and Monte Carlo methods[1–14].

Previously, we developed an analytical model for estimating terrestrial cosmic-ray fluxes anywhere in Earth’s atmosphere at nearly any given time [15–17] by modeling the results of an EAS simulation performed using the Particle and Heavy Ion Transport Code System (PHITS) [18]. The model comprised several theoretical or empirical equations with free parameters, whose numerical values were determined from the least square (LSq) fitting of EAS data. Omnidirectional fluxes of neutrons, protons, ions with a charge of up to 28 (Ni), muons, electrons, positrons, and photons could be calculated using the model over an energy range of 10 keV–1 TeV (per nucleon for ions), with the exception of neutrons, for which fluxes could be calculated down to 0.01 eV. The model was named PHITS-based Analytical Radiation Model in the Atmosphere (PARMA) and was implemented through the open-access EXcel-based Program for calculating Atmospheric Cosmic-ray Spectrum (EXPACS) [19]. PARMA and EXPACS have been extensively used in various research fields, including radiation protection [20–22], semiconductor design [23,24], and the geosciences [25–29].

In addition to the omnidirectional fluxes, their angular distributions are also requested to be evaluated in some applications. For example, the estimation of muon fluxes, particularly in the horizontal direction, is useful in the planning of muon radiography of large objects such as volcanoes [30,31], nuclear reactors [32,33], and pyramids [34]. Accurate modeling of the generation of terrestrial cosmic rays considering their angular distributions is the key issue in the Monte Carlo simulation used in designing cosmic-ray detectors [35], shielding calculation for electric devices [36], and soil moisture monitoring [37]. However, there is no model that can instantaneously calculate angular differential cosmic-ray fluxes in the atmosphere for all conditions. The cosmic-ray shower library, CRY [38], can generate cosmic-ray particle shower distribution for use as input to such Monte Carlo simulations, but it is applicable only to three discrete elevations (sea level, 2,100 m, and 11,300 m).

With such situations in mind, we extended our previously established model, PARMA3.0, to make it capable of calculating not only omnidirectional but also angular differential terrestrial cosmic-ray fluxes. This extended version was designated PARMA4.0. The results of EAS simulation performed in our previous study [17] were employed to improve the model, and we carried out additional EAS simulations by changing the ground conditions in order to investigate the effect of Earth’s albedo on cosmic-ray angular distributions. A brief outline of the procedures of the EAS simulation together with analysis of the obtained angular distributions are given in the section titled “EAS Simulation”, details on the extended capability of PARMA are described in the section titled “Development of PARMA4.0”, and comparisons of the results obtained from PARMA4.0 with those from the EAS simulation and several experiments are presented in the section titled “Verification of PARMA4.0”. The final section presents our concluding remarks.

## EAS Simulation

### Simulation Procedure

The procedure for our EAS simulation is described in detail in our previous paper [17], and therefore only a brief description is given in this paper. The atmosphere is divided into 28 concentric spherical shells, and its maximum altitude is assumed to be 86 km. The densities of each shell are determined by referring to US Standard Atmosphere 1976. The Earth is represented as a sphere with a radius of 6,378.14 km, and its composition is presumed to be the same as that of the air at sea level in order to obtain terrestrial cosmic-ray fluxes under the ideal condition, i.e., without disturbance from the ground. This ground condition is referred to “ideal atmosphere” in this paper.

In the EAS simulation, cosmic rays are incident from the top of the atmosphere in the isotropic irradiation geometry. GCR protons and heavy ions with energies and charges of up to 1 TeV/n and 28 (Ni), respectively, were considered as source particles. The energy spectra of GCR were determined by the model proposed by Matthiä et al. [39]. EAS simulations were conducted for near solar minimum and maximum conditions, i.e., *W* = 0 and 150, respectively, and for 21 geomagnetic field locations with vertical cut-off rigidities *r*_c_ ranging from 0 to 20 GV. Note that the solar activity index, *W*, roughly represents the sun spot number, but its numerical values were determined from the count rates of several neutron monitors in our study. Note that the trapped particles as well as reentrant albedo particles were not taken into account as the source particles in the simulation.

The atmospheric propagation of incident cosmic rays and their associated EAS were simulated using PHITS version 2.73. Default nuclear reaction models and data libraries adopted in PHITS2.73, such as INCL4.6 [40], were employed in the simulation, except in the total reaction cross section model, which was particularly adjusted for high-energy particle transport simulations [41]. In the simulation, all particles were traced down to 10 keV, with the exception of neutrons, which were transported down to 0.01 eV. A variance reduction technique was adopted for transporting low-energy electrons, positrons, and photons in order to reduce the computational time. The angular distribution of particles crossing 18 surfaces at altitudes of between 0 and 52 km were scored as a function of cos(*θ*), where *θ* is the zenith angle to the downward direction. As shown in our previous paper [17], the accuracy of the EAS simulation was carefully confirmed using experimental omnidirectional flux data for neutrons and vertical downward flux data for the other particles.

In order to investigate the effect of Earth’s albedo on cosmic-ray angular distributions, we conducted two additional EAS simulations that involved changing the composition of Earth: one simulation employed a realistic ground surface, while the other featured a virtual black hole that absorbed all radiation, i.e., it had no albedo effect. The composition of the realistic ground surface was assumed to be 60% SiO_2_, 20% Al_2_O_3_, and 20% H_2_O by mass. These simulations were performed for only the polar region at the solar minimum condition, i.e., at *W* = 0 and *r*_c_ = 0 GV, and only the angular distributions at the ground surface were scored. The results obtained from the virtual black hole condition can be used in source generation for underground cosmic-ray transport simulation, which is useful in applications such as soil moisture monitoring with neutrons [37].

### Results of the EAS Simulation

**Fig 1:**
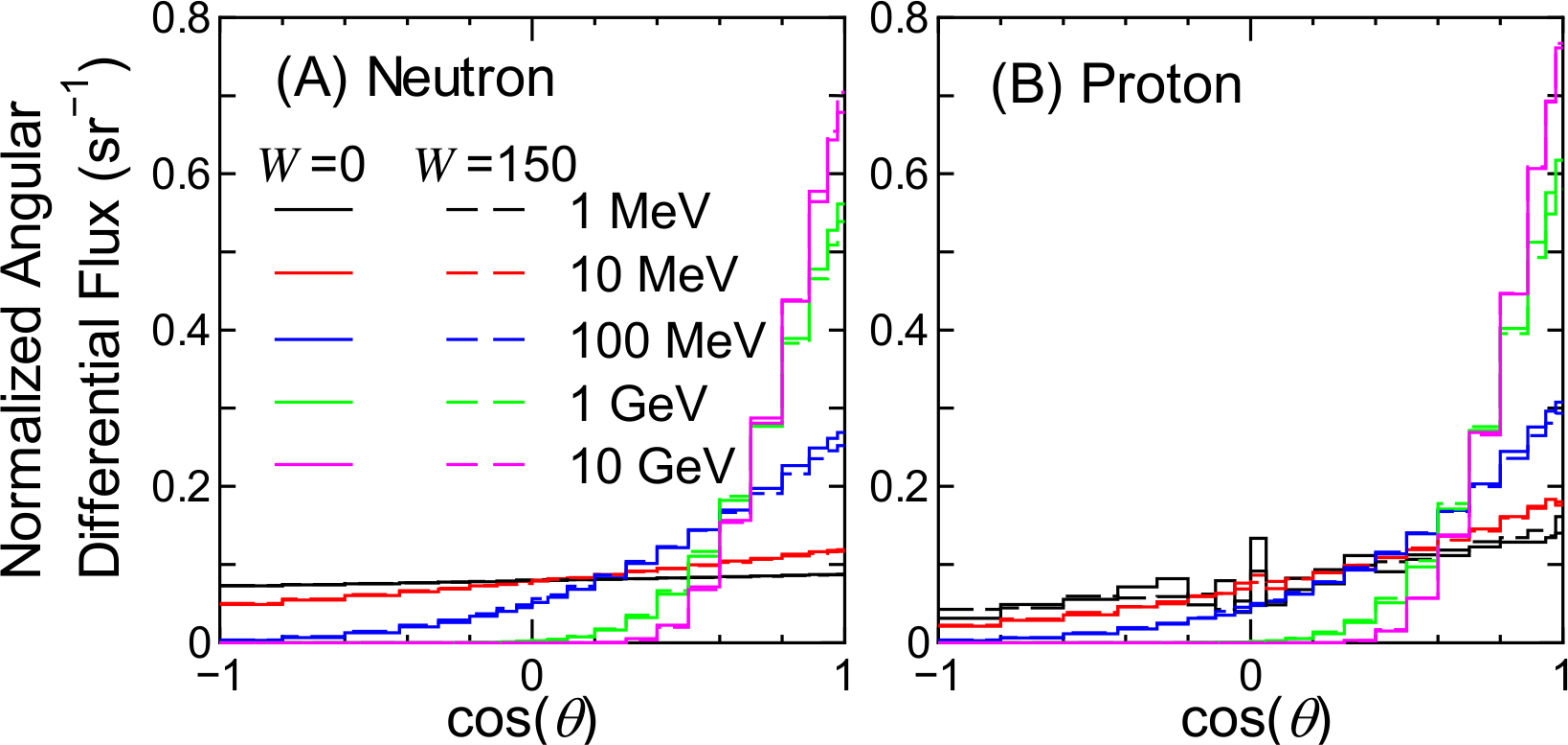
Angular distributions of neutron and proton fluxes obtained from EAS simulation for the solar minimum and maximum conditions at the altitude of 8 km and *r*_c_ = 0 GV.

In this subsection, the angular distributions of terrestrial cosmic-ray fluxes for various conditions are presented in units of sr^-1^, which are to be determined by the analytical model proposed in the next section. Note that cos(*θ*) = 1 indicates the vertical downward direction. The integral of the angular differential fluxes is normalized to 1.0. Fig 1 shows examples of angular distributions for neutron and proton fluxes for the solar minimum and maximum conditions. It is evident from the figure that the angular distributions for the two solar conditions are nearly identical to each other. Similar tendencies are also seen for other particles and condition cases, indicating that the angular distributions of terrestrial cosmic-ray fluxes are almost independent of the solar activity. We therefore adopt the mean angular distributions between the solar minimum and maximum conditions in the further analysis presented in this paper.

**Fig 2:**
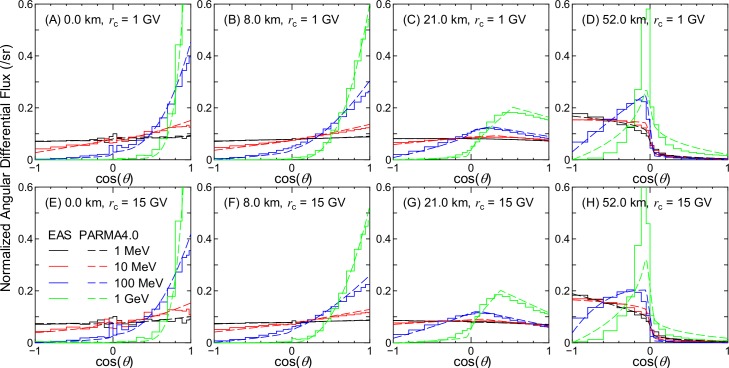
Angular distributions of neutron fluxes obtained from EAS simulation and PARMA4.0.

**Fig 3:**
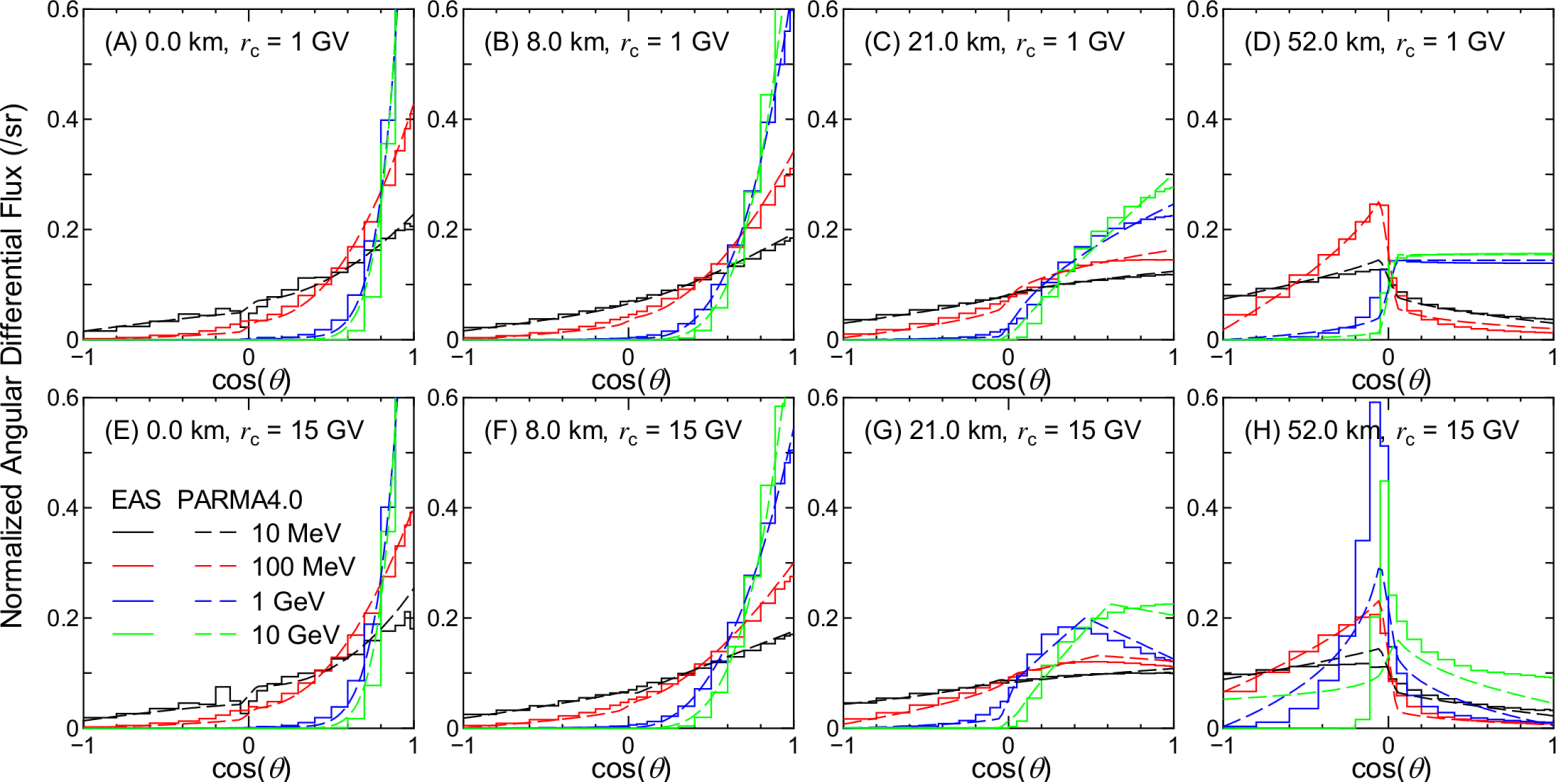
Angular distributions of proton fluxes obtained from EAS simulation and PARMA4.0.

**Fig 4:**
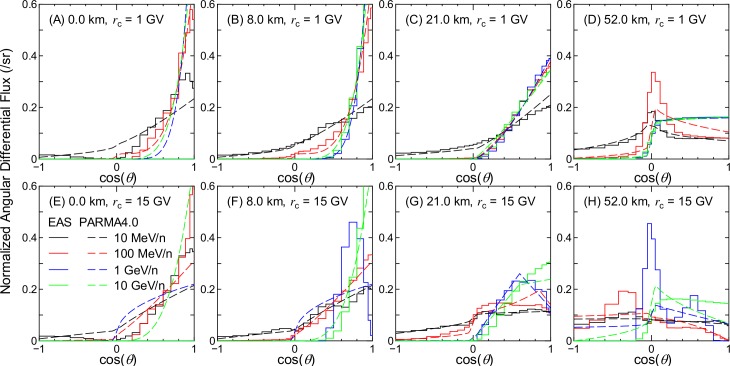
Angular distributions of He ion fluxes obtained from EAS simulation and PARMA4.0.

**Fig 5:**
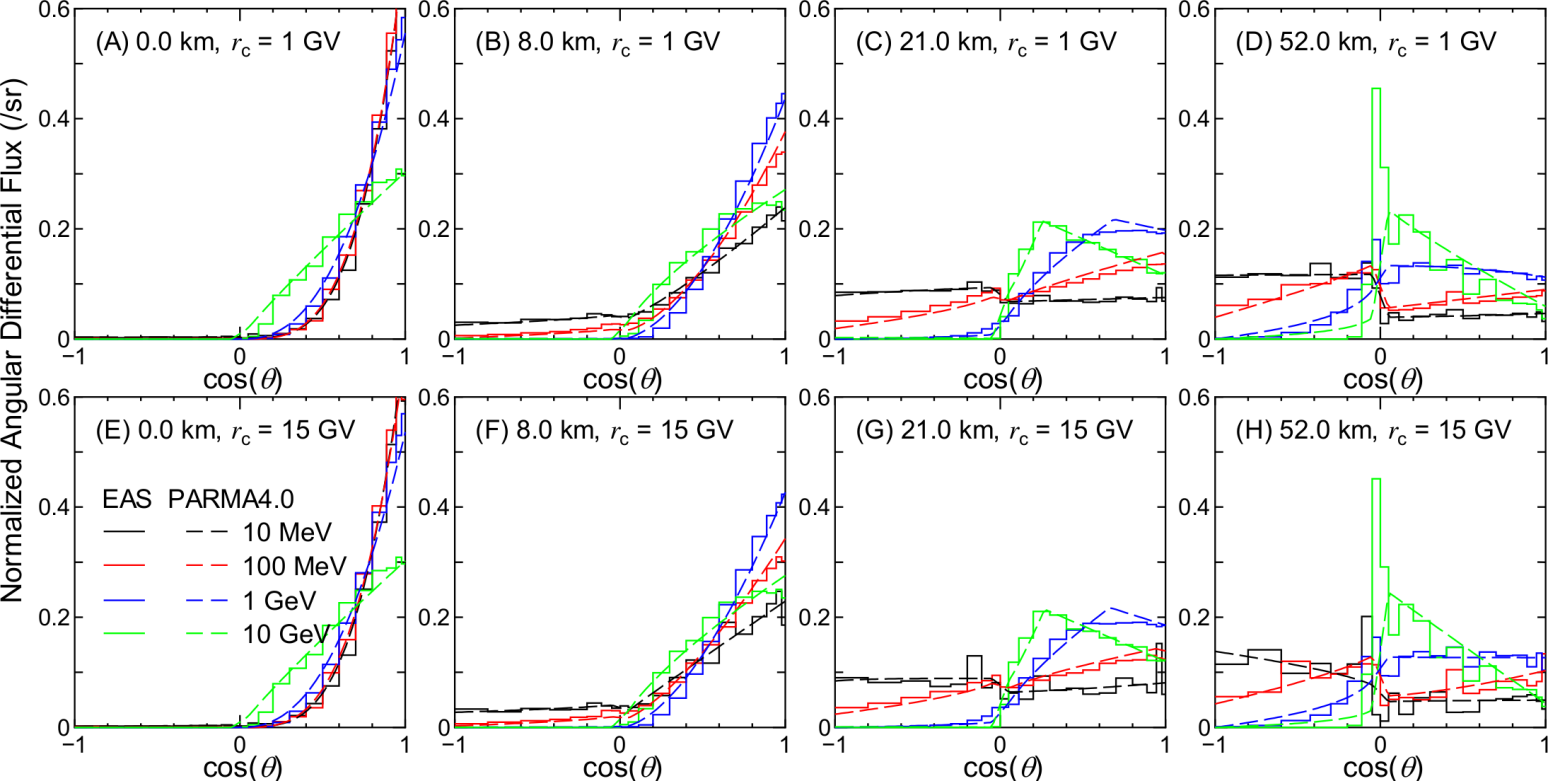
Angular distributions of muon fluxes obtained from EAS simulation and PARMA4.0.

**Fig 6:**
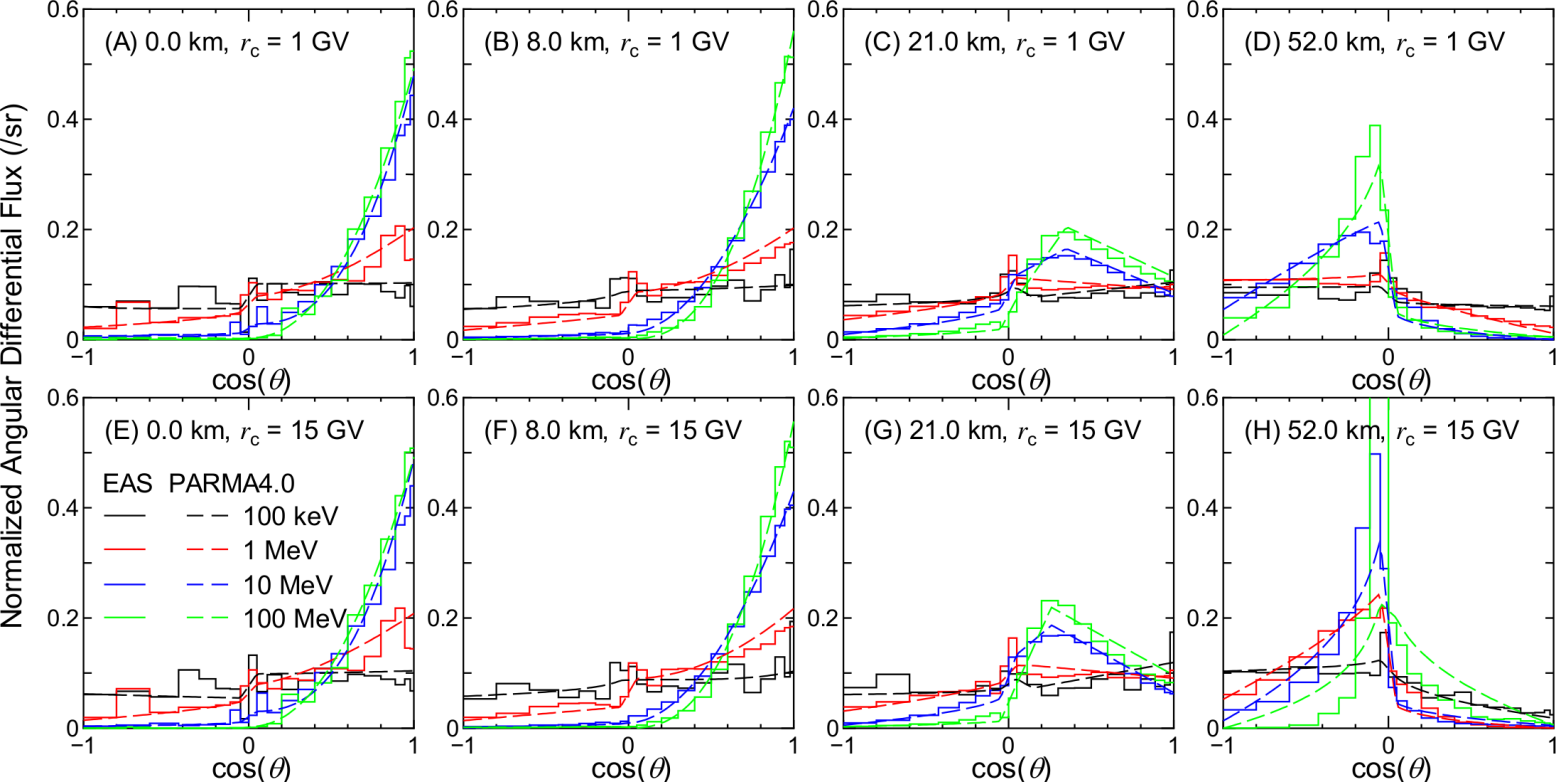
Angular distributions of the sum of electron and positron fluxes obtained from EAS simulation and PARMA4.0.

**Fig 7:**
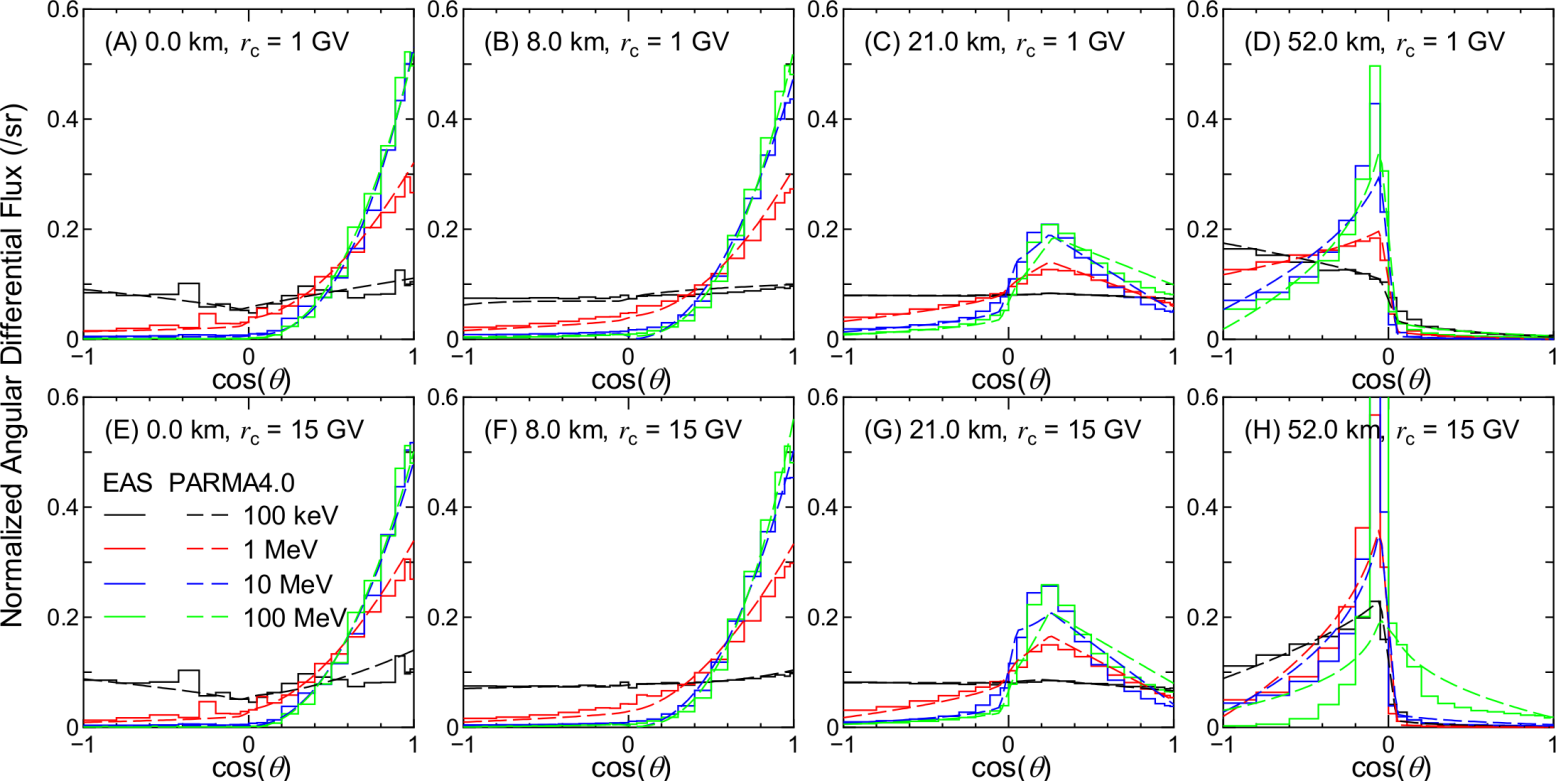
Angular distributions of photon fluxes obtained from EAS simulation and PARMA4.0.

Figs 2–7 show examples of angular distributions for neutrons, protons, He ions, μ^±^, e^±^, and photons, respectively, for various altitudes at *r*_c_ = 1 and 15 GV. The results obtained from the analytical model proposed in the next section, PARMA4.0, are also shown in the figures. As the angular distributions of electron and positron fluxes are very similar to each other, they are not distinguished in the analysis presented in this paper. The statistical uncertainties of the EAS data are less than 20% in most cases, but they become larger in some situations, particularly around cos(*θ*) = 0, because particle fluxes were calculated based on the number of particles passing through a surface divided by cos(*θ*) in the EAS simulation. The statistical uncertainties of the He ion data are also very large at locations where the primary ions cannot penetrate, e.g., no high-energy He ions are observed at sea level.

It is evident from the figures that the angular distributions vary significantly with altitude, but they are not as sensitive to the vertical cut-off rigidity, except in the cases of high-energy protons and He ions at high altitudes. It is also seen that most EAS data cannot be expressed on the basis of simple equations such as cos^*n*^(*θ*) that are frequently used for representing the angular distributions of high-energy cosmic-rays at ground level. At lower altitudes, the angular distributions for high-energy particles exhibit strong downward directivity and become closer to isotropic with decreasing energy. With an increase in altitude, the downward directivity becomes less significant, and the angles of peak flux gradually shift toward the horizontal direction. This tendency can be explained in terms of the Pfotzer maximum, which arises because primary cosmic rays must travel a certain distance through the atmosphere in order to fully form a cascade of secondary particles; as the mean distance that cosmic rays travel at a given altitude becomes longer with decreasing cos(*θ*), the peak flux correspondingly shifts toward the horizontal at high altitudes. Conversely, the angular distributions for particles dominantly composed of primary cosmic rays, such as protons and He ions over 1 GeV/n at 52 km and 1 GV, are nearly constant between 0 < cos(*θ*) < 1, as shown in panel (D) of Figs 3 and 4. The primary and secondary cosmic rays have a dominant contribution only at lower and higher *r*_c_, respectively, and this is the reason why the angular distributions for the proton and He ion data at high altitudes significantly depend on the vertical cut-off rigidity.

**Fig 8:**
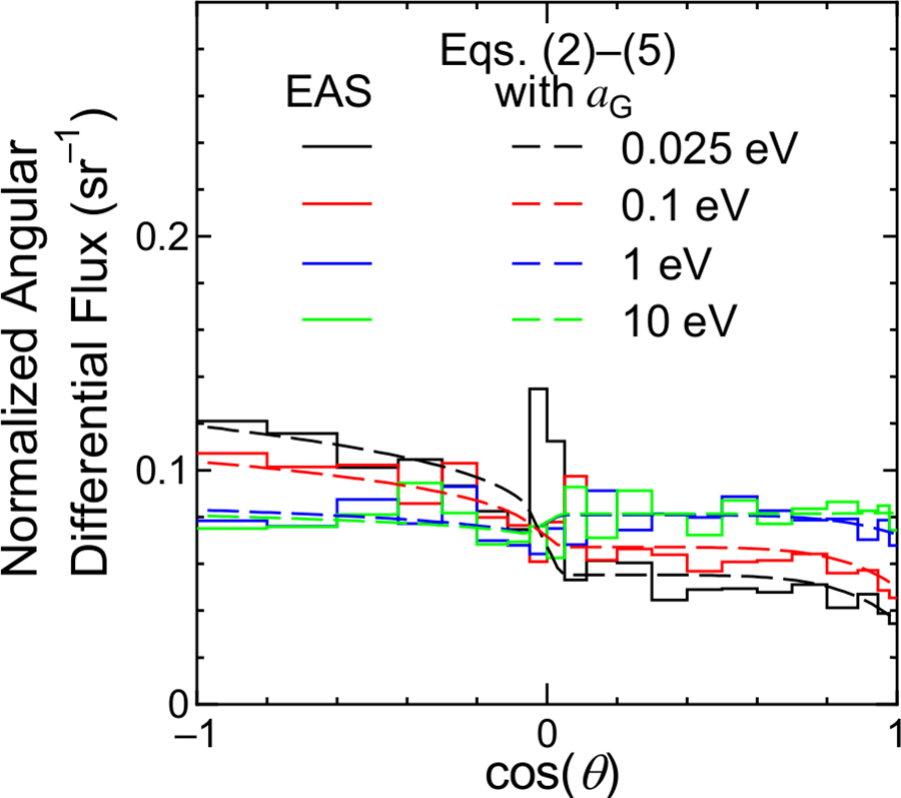
Angular distributions of low-energy neutron fluxes obtained from EAS simulation employing a realistic ground surface compared to corresponding data calculated by PARMA4.0.

Fig 8 shows examples of the angular distributions of low-energy neutron fluxes at sea level obtained from EAS simulation employing a realistic ground surface. The peak observed around cos(*θ*) = 0 is probably caused by large statistical uncertainties of the EAS data in the horizontal direction. The results calculated using the equations proposed in the next section are also shown in the figure. It is seen that the upward fluxes are slightly larger than the downward fluxes at the thermal energy, i.e., 0.025 eV, while the angular distributions are almost isotropic for energies above 1 eV; this is because the neutrons tend to be thermalized in ground as suggested in our previous study [15]. Note that the ground surface has little influence on the angular distributions for higher energy neutrons as well as for other particles.

**Fig 9:**
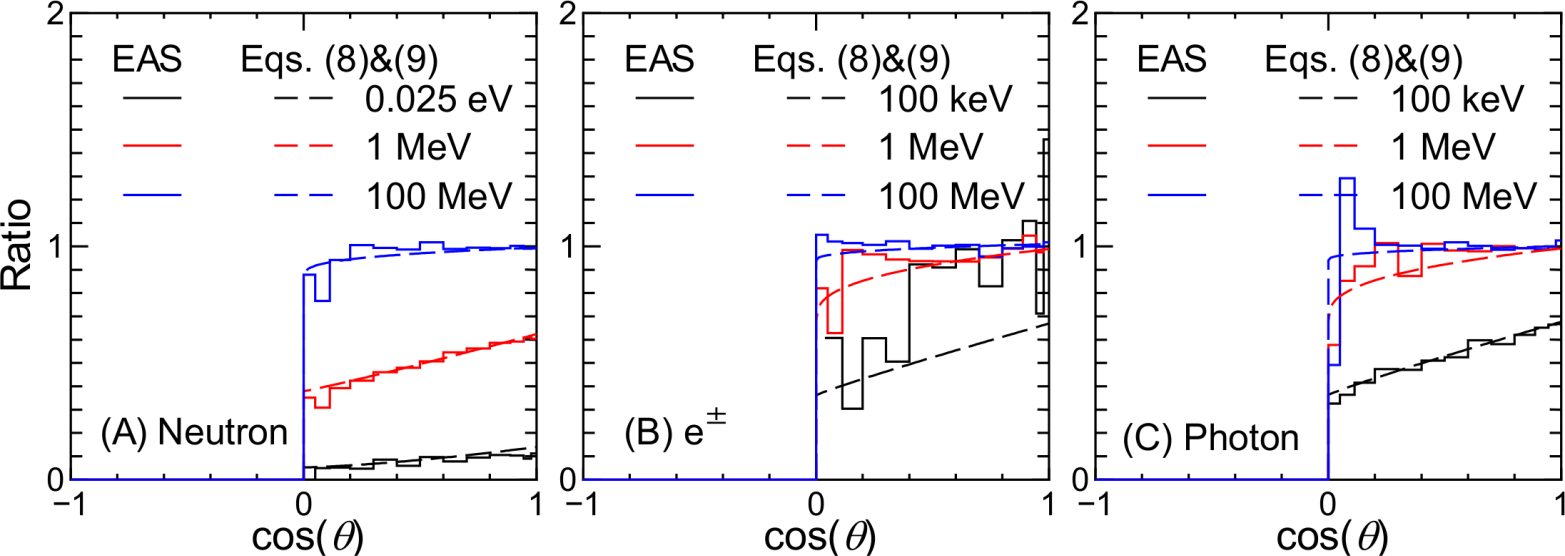
Ratio between particle fluxes at sea level obtained from EAS simulations employing the virtual black hole and the ideal atmosphere as the Earth.

Fig 9 shows the ratios of particle fluxes at sea level between the EAS simulation results obtained employing the virtual black hole and those obtained using the ideal atmosphere as the Earth. The ratio is equal to 0.0 in the upward direction, i.e., cos(*θ*) < 0, because there are no albedo particles from the virtual black hole. In the downward direction, the ratios become larger (and asymptotic to 1.0) with increasing energy and cos(*θ*). As indicated in the figure, these data were also reproduced by the equations proposed in the next section.

## Development of PARMA4.0

### General Description of PARMA4.0

PARMA4.0 (PHITS-based Analytical Radiation Model in the Atmosphere, Version 4.0) facilitates instantaneous estimation of both omnidirectional and angular differential terrestrial cosmic-ray fluxes of neutrons, protons, He ions, muons, electrons, positrons, and photons at nearly any time and any place in the Earth’s atmosphere. The omnidirectional fluxes of ions with a charge of up to 28 (Ni) can be also calculated in the same manner as in PARMA3.0. The unit of the output flux is either cm^−2^s^−1^MeV^−1^ or cm^−2^s^−1^MeV^−1^sr^-1^, with input parameters including atmospheric depth *d* in g/cm^2^, vertical cut-off rigidity *r*_c_ in GV, solar modulation index *W*, kinetic energy *E* in MeV, and the zenith angle *θ*, with the last factor required only for calculating angular differential fluxes. Note that the ion fluxes are output in cm^−2^s^−1^(MeV/n)^−1^sr^-1^ or cm^−2^s^−1^(MeV/n)^−1^ and their kinetic energies should be given in MeV/n.

In PARMA4.0, the angular differential flux of terrestrial cosmic rays, *φ*, is expressed as the product of their omnidirectional flux in the ideal atmosphere, *ϕ*_omni_, the correction factor for local geometry effects, *f*_L_, and the normalized angular distribution, *Φ*, as given by

$$\varphi (x,E,d,r_{\text{c}},W,g)=\phi_{\text{omni}}\left(E,d,r_{\text{c}},W\right)f_{\text{L}}\left(g,E\right)\Phi \left(x,E,d,r_{\text{c}}\right),$$ (1)

where *x* is cos(*θ*) and *g* is the local geometry parameter, e.g., water density in the ground or aircraft mass, as described in [15]. The values of *ϕ*_omni_ and *f*_L_ were obtained from a slightly revised version of PARMA3.0, while *Φ* was calculated using mathematical functions proposed later in this section. We assumed that *Φ* was independent of the solar activity, *W*, based on the discussion given in the previous section. Note that the azimuth angle dependence is not modeled in PARMA4.0 because the east-west effect was not considered in our EAS simulation.

The particle energies covered by PARMA4.0 are 10 keV–1 TeV (per nucleon for ions), except for neutrons and muons, whose fluxes can be calculated down to 0.01 eV and up to 100 TeV, respectively. However, the calculated fluxes for neutrons, e^±^, and photons with energies above approximately 5 GeV are not precise enough for the proposed method, as will be discussed later. In terms of global conditions, the applicable altitude, geomagnetic, and temporal ranges of PARMA4.0 are from sea level to the top of the atmosphere, from the polar region to the equatorial region, and from minimum to maximum solar activities recorded in the last 400 years, respectively. Note that the normalized angular distributions are assumed to remain constant above 52 km, the altitude up to which angular differential fluxes were scored in our EAS simulation.

### Normalized Angular Distributions in the Ideal Atmosphere

As shown in Figs 2–7, the shapes of the normalized angular distributions are so complicated that they cannot be expressed by a simple function. For example, sudden increases or decreases of the fluxes are observed at cos(*θ*) = 0, particularly in the data for higher altitudes; the high-energy fluxes reach their maximum level at an intermediate angle around cos(*θ*) = 0.5 at the altitude of 21 km. We therefore divided the angular distributions into four components, namely upward, horizontal, downward, and extra-downward fluxes, and expressed them as follows:

$$\Phi (x,E)=a_{1}\left(E\right)+a_{2}\left(E\right){\left[-x\right]}^{a_{3}\left(E\right)}\;\text{for}\;–1\leq x\leq x_{\text{UH}},$$ (2)

$$\Phi (x,E)=\frac{\Phi (x_{\mathrm{UH}},E)x_{\mathrm{HD}}-\Phi (x_{\mathrm{HD}},E)x_{\mathrm{UH}}}{x_{\mathrm{HD}}-x_{\mathrm{UH}}}+\frac{\Phi (x_{\mathrm{HD}},E)-\Phi (x_{\mathrm{UH}},E)}{x_{\mathrm{HD}}-x_{\mathrm{UH}}}x\;\text{for}\;x_{\text{UH}}<x<x_{\text{HD}},$$ (3)

$$\Phi (x,E)=a_{4}\left(E\right)+a_{5}\left(E\right)x^{a_{6}\left(E\right)}\;\text{for}\;x_{\text{HD}}\leq x\leq x_{\text{DE}},$$ (4)

$$\Phi (x,E)=\frac{\Phi (x_{\mathrm{DE}},E)-a_{7}\left(E\right)x_{\mathrm{DE}}}{1-x_{\mathrm{DE}}}+\frac{a_{7}\left(E\right)-\Phi (x_{\mathrm{DE}},E)}{1-x_{\mathrm{DE}}}x\;\text{for}\;x_{\text{DE}}<x\leq 1,$$ (5)

where *Φ*(*x*,*E*) is the normalized angular distribution in the ideal atmosphere at cos(*θ*) = *x* for a particle with energy *E*, the parameters *x*_UH_, *x*_HD_, and *x*_DE_ represent the switching angles from upward to horizontal, horizontal to downward, and downward to extra-downward fluxes, respectively, and *a*_1_ to *a*_7_ are free parameters. Both *Φ* and *a*_*i*_ depend not only on *E*, but also on *d* and *r*_c_, although their dependencies are not explicitly denoted in these equations because they are not continuous functions of *d* and *r*_c_. The numerical values of *a*_*i*_ were determined from LSq fitting to the EAS data for each energy, altitude, and vertical cut-off rigidity. However, the values for He ions below an altitude of 8 km were assumed to be the same as those at 8 km, owing to significantly high statistical uncertainties of the EAS data, as shown in Fig 4.

For lower altitudes at which *Φ* smoothly and continuously increases from the upward to the downward direction, Eq (3) and Eq (5) are not necessary, and the numerical values of *a*_1_ and *a*_4_ should be identical. At around cos(*θ*) = 0, however, *Φ* dramatically changes for higher altitudes and thus is simply determined from the linear interpolation of *Φ*(*x*_UH_,*E*) and *Φ*(*x*_HD_,*E*) obtained from Eqs (2) and (4), respectively, in our model. The numerical values of *x*_UH_ and *x*_HD_ were fixed at –0.05 and 0.05, respectively, for all conditions. For the case in which a peak is observed at an intermediate angle of approximately cos(*θ*) = 0.5, *Φ* above the peak angle is also determined from the linear interpolation of *Φ*(*x*_DE_,*E*) and *a*_7_, where the values of *x*_DE_ and *a*_7_ are fitted to reproduce the peak angle and *Φ*(1.0,*E*), respectively, obtained from the EAS simulation. Thus, *x*_DE_ is regarded as a free parameter *a*_8_, unlike the fixed *x*_UH_ and *x*_HD_. Note that the value of *a*_8_ is equal to 1.0 for most cases, and, therefore, Eq (5) is not used for calculating *Φ* for those conditions.

**Fig 10:**
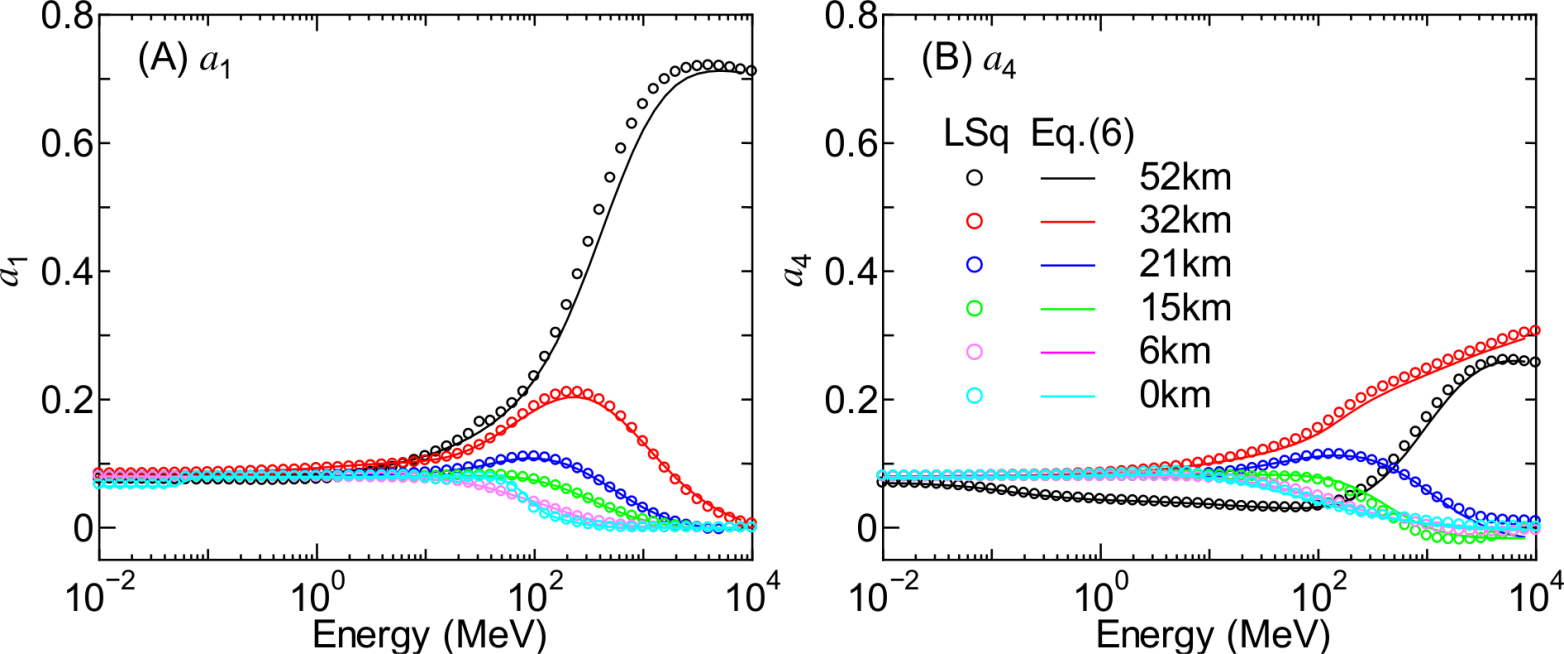
Numerical values of *a*_1_ and *a*_4_ for neutrons at *r*_c_ = 1 GV for various altitudes determined by LSq fitting and calculated by Eq (6).

As examples of the results from the LSq fitting, Fig 10 shows the evaluated *a*_1_ and *a*_4_ for neutrons at *r*_c_ = 1 GV for various altitudes as a function of energy. In order to obtain smooth curves, the EAS data were averaged over nearly one decade of energy in the LSq fitting, e.g., the *a*_1_ value for 1 MeV was determined from the mean of the EAS data between approximately 0.3 and 3 MeV. The evaluated *a*_1_ and *a*_4_ are asymptotic to 1/4π with decreasing energy, indicating the isotropic distribution for low-energy neutrons. The values of *a*_1_ and *a*_4_ are close to each other except at higher altitudes, where sudden increases or decreases of the fluxes are observed in the horizontal direction.

**Fig 11:**
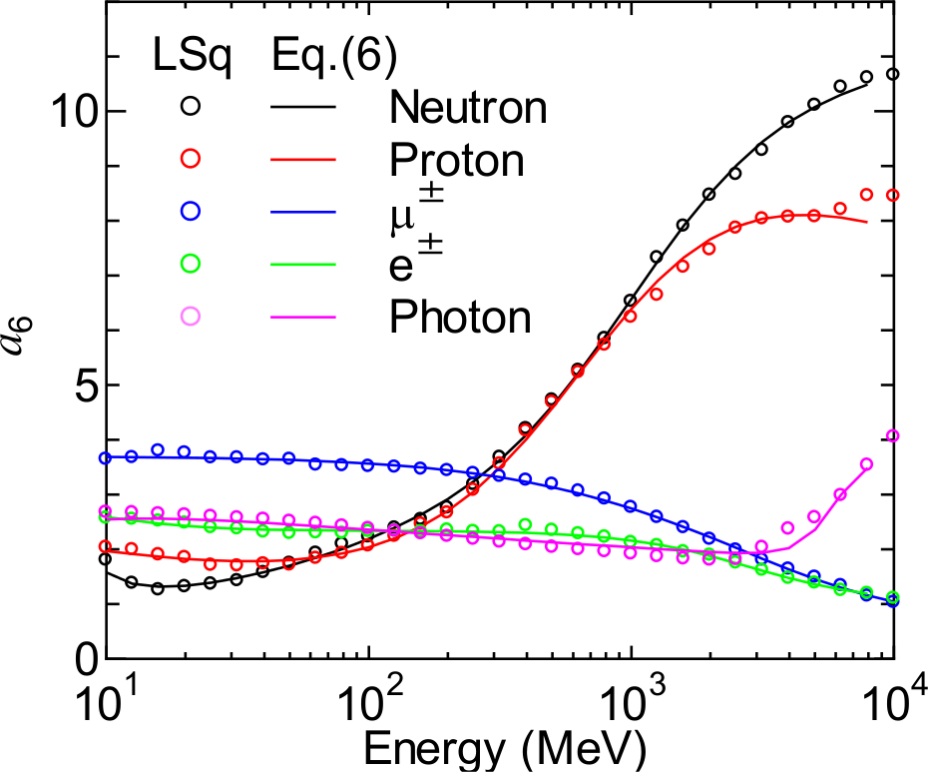
Numerical values of *a*_6_ for neutrons, protons, μ^±^, e^±^, and photons at sea level and *r*_c_ = 1 GV determined by LSq fitting and calculated by Eq (6).

Fig 11 shows the evaluated *a*_6_ parameters for neutrons, protons, μ^±^, e^±^, and photons above 10 MeV at sea level and *r*_c_ = 1 GV. The angular distributions for these conditions can be approximated by a frequently used function of cos^*n*^(*θ*), and thus *a*_6_ is a rough indication of *n*. It is seen from the graph that the *a*_6_ parameters for neutrons and protons increase with energy, while the relation is reversed for other particles. The reason for decreasing *a*_6_ for high-energy leptons is that they are predominantly generated through extremely high-energy muons, whose angular distributions are nearly isotropic or even increasing in the horizontal direction, as discussed later. Note that the sudden increase in the *a*_6_ parameter for high-energy photons is probably caused by the poor statistics of the EAS data owing to the very small fluxes of such high-energy photons.

One of the fundamental development strategies of PARMA is to reduce the number of free parameters as much as possible. We therefore express the energy dependences of the parameters *a*_1_ to *a*_8_ as the sum of three sigmoid functions:

$$a_{i}\left(E\right)=b_{i,1}+\sum_{k=1,3}\frac{b_{i,3k-1}}{1+\text{exp}\left\{\left[b_{i,3k}-\log_{10}\left(E\right)\right]/b_{i,3k+1}\right\}},$$ (6)

where *b*_*i*,1_ to *b*_*i*,10_ are free parameters determined for each altitude and vertical cut-off rigidity where EAS data are available. This equation is introduced only to reproduce the complicated energy dependences of *a*_i_, and its form has little physical meaning. In the cases where *a*_*i*_ has a relatively simple energy dependence, the second and/or third sigmoid functions are not necessary, i.e., *b*_*i*,5_ and/or *b*_*i*,8_ = 0. The numerical values of *b*_*i*,*j*_ were determined from LSq fitting to the evaluated *a*_*i*_ parameters, with the results of the fitting shown in Figs 10 and 11. It is evident from the figures that Eq (6) can reproduce the data very well.

There are, however, two problems in the calculation of *a*_*i*_ using Eq (6). When upward fluxes are much smaller than downward fluxes, as is the case for high-energy particles at sea level, the value of *a*_2_ calculated by Eq (6) is occasionally smaller than (–*a*_1_), and, consequently, *Φ*(–1.0,*E*) calculated by Eq (2) becomes negative. In such cases, the *a*_2_ value is adjusted to produce a positive *Φ*(–1.0,*E*), though this adjustment sometimes induces a discontinuity in the energy spectrum. Similar adjustments are also necessary for *a*_5_ when the downward fluxes are much smaller than the upward fluxes. The other problem is the necessity of renormalization of *Φ*. The integral of *Φ*(*x*,*E*) with respect to *x* between –1 and 1 should be equal to 1/2π, but this is not true when the *a*_*i*_ calculated by Eq (6) are directly substituted into Eqs (2) to (5). Thus, the angular distributions must be renormalized by multiplying by the ratio of 1/2π and the original integral value of *Φ*.

In contrast to energy, the altitude and vertical cut-off rigidity dependences of *b*_*i*,*j*_ are so complex that they cannot be expressed in simple form. Thus, we evaluated *b*_*i*,*j*_ for 18 altitudes and 7 vertical cut-off rigidities and determined *Φ* for intermediate conditions by simply interpolating *Φ* where the evaluated *b*_*i*,*j*_ were available.

The calculated angular distributions are shown in Figs 2–7. The agreements between the EAS and PARMA4.0 results are generally satisfactory, except for the cases in which the EAS data are significantly fluctuated owing to large statistical uncertainties such as those in the He ion data at sea level. Disagreements are also observed when the EAS data have a sharp peak in the horizontal direction, as is true for the high-energy neutron data at 52 km. This occurs because the angular distributions between –0.05 < cos(*θ*) < 0.05 are simply interpolated using Eq (3) in PARMA4.0. A more comprehensive comparison between the EAS and PARMA4.0 data will be provided in the next section.

### Correction for the Ground and Black Hole Effects

As shown in Fig 8, the presence of ground changes only the angular distributions of neutrons around the thermal energy. In order to express this change, we introduced the corrected *a* parameter, *a*_G_, for *a*_1_, *a*_2_, *a*_4_ and *a*_5_ as given by:

$$a_{\text{G},i}\left(E\right)=a_{i}\left(E\right)+\frac{c_{i,1}}{1+\text{exp}\left\{\left[\log_{10}\left(E\right)-c_{i,2}\right]/c_{i,3}\right\}},$$ (7)

where *c*_*i*,1_ to *c*_*i*,3_ are free parameters determined from LSq fitting to the EAS data at sea level for the ground condition. The evaluated values of *c*_*i*,2_ and *c*_*i*,3_ are around –7.0 and 0.5, respectively, and thus, the values of *a*_G_,_*i*_ are nearly equal to *a*_*i*_ for neutrons with energies above approximately 1 eV because of a large value of exp{[log_10_(*E*)−*c*_*i*,2_]/*c*_*i*,3_}. This result confirms that it is not necessary to consider the ground effect in the calculation of neutron angular distributions except around the thermal energy. Replacing *a*_*i*_ with *a*_G_,_*i*_ in Eqs (2) to (5), the normalized angular distributions for neutron fluxes at the ground level can be calculated. The results of these calculations are shown in Fig 8, from which it is evident that the normalized angular distributions obtained from the EAS simulation and Eqs (2) to (5) with *a*_G_,_*i*_ agree very closely.

For calculating the angular differential fluxes incident to the virtual black hole, the correction factor for the local geometry effects, *f*_L_(*g*,*E*), in Eq (1) should be replaced by that for the virtual black hole effect, *f*_B_(*x*,*E*). This correction factor can be determined from the ratio of particle fluxes at sea level obtained from EAS simulations respectively employing the virtual black hole and the ideal atmosphere as the Earth, examples of which are shown in Fig 9. To express *f*_B_(*x*,*E*), we propose the following equations:

$$f_{\text{B}}(x,E)=0\;\text{for}–1\leq x<0,$$ (8)

$$f_{\text{B}}(x,E)=a_{9}\left(E\right)+\left[a_{10}\left(E\right)-a_{9}\left(E\right)\right]x^{a_{11}(E)}\;\text{for}\;0\leq x\leq 1,$$ (9)

where *a*_9_ to *a*_11_ are free parameters determined from LSq fitting to the EAS data. The *a*_9_ and *a*_10_ parameters represent *f*_B_(0,*E*) and *f*_B_(1,*E*), respectively, and they are asymptotic to 1.0 with increasing *E*. The energy dependences of *a*_9_ to *a*_11_ are also expressed by Eq (6).

The calculated *f*_B_ based on the evaluated *a*_*i*_ parameters are shown in Fig 9. It is evident that Eqs (8) and (9) can reproduce the EAS data satisfactorily, although some discrepancies are observed owing to large statistical uncertainties in the EAS data, particularly for low-energy electrons and positrons. Note that the *a*_9_ to *a*_11_ parameters for protons, He ions, and muons are assumed to be 1.0 because most albedo-charged particles other than electrons and positrons do not return to the ground owing to their small scattering cross sections at large angles.

### Correction for Muon Fluxes

**Fig 12:**
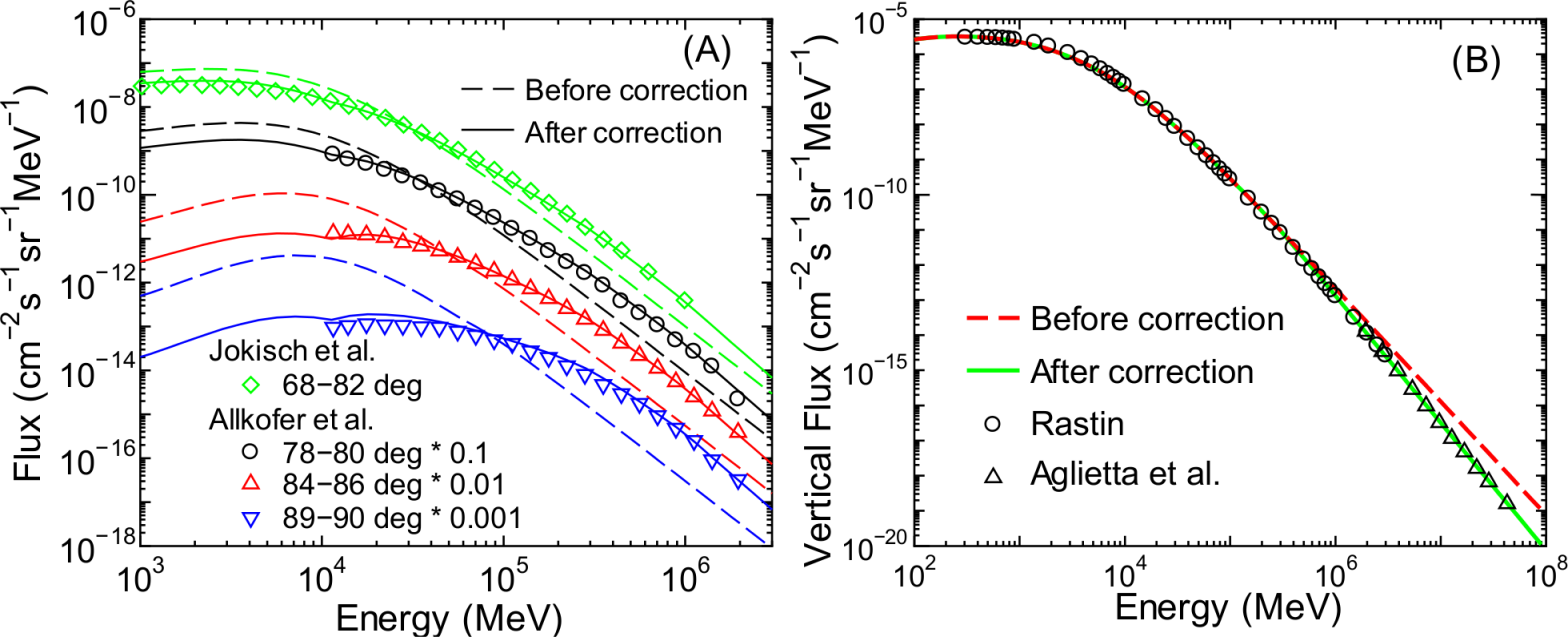
**Angular differential energy spectra of muon fluxes at ground level for (A) large zenith angles [42,43] and (B) the vertical direction [44, 45] obtained from experiments and our calculations before and after muon flux corrections**.

Estimation of the angular differential energy spectra of high-energy muon fluxes at ground level, particularly in the horizontal direction, is of great importance in the planning of muon radiography. However, as shown in Fig 12, the model proposed above cannot reproduce the measured muon fluxes at ground level for large zenith angles [42,43] or even in the vertical direction [44,45] when the muon energy is over 100 GeV. This is because such muons are generally produced by primary cosmic rays with energies over 1 TeV/n, which are not considered in our EAS simulation. The predictability of such high-energy muon fluxes is crucial in the model for applying to the design of muon radiography, and thus, we introduce the correction factors to fix the discrepancy.

The discrepancy observed in high-energy muon fluxes for the vertical direction is attributable to the inaccuracy of PARMA3.0 calculating their omnidirectional fluxes instead of their angular distributions, as the model was designed to reproduce the EAS data up to around 100 GeV. The power index for expressing high-energy muon spectra at sea level used in PARMA3.0 is –3.23, which is much larger than the values obtained from experimental data [44,45]. Thus, we revised PARMA3.0 to reduce the power index of muons with energy above a certain threshold, *E*_t_, and normalized the higher energy muon fluxes by $E_{\text{t}}^{\gamma_{\text{d}}}$, where γ_d_ is the difference between the power indices below and above *E*_t_. To reproduce the experimental data, the numerical values of *E*_t_ and γ_d_ were determined to be approximately 30 GeV and 0.4, respectively. The vertical muon fluxes calculated using high-energy correction are shown in panel (B) of Fig 12. Excellent agreement is seen between the measurements and the revised calculations, even for energies above 100 GeV. Owing to this correction, the applicable energy of muons covered by PARMA4.0 is extended up to 100 TeV. Note that this correction is considered in calculating the omnidirectional muon fluxes not only at ground level but also at higher altitudes.

**Fig 13:**
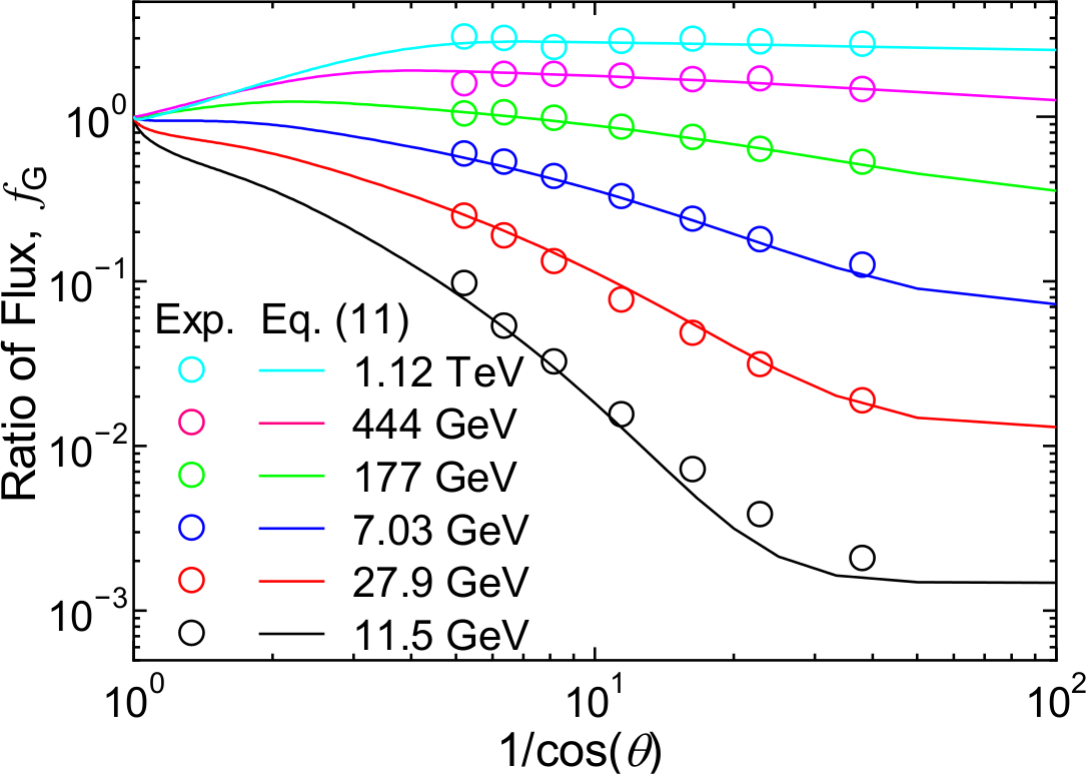
Ratio between muon fluxes at a specific zenith angle θ to those in the vertical direction, *f*_G_, as a function of 1/cos(*θ*). Dots are determined from the ratio between measured fluxes [43] and PARMA4.0-calculated vertical fluxes, while lines are calculated by Eq (11).

On the other hand, the discrepancies observed in muon fluxes for large zenith angles are attributed to the inaccuracy of calculating their angular distributions, particularly at ground level. Considering that muon vertical fluxes can be accurately reproduced by our model, we correlated the vertical fluxes to those in other directions on the basis of experimentally determined angular distributions at ground level:

$$\varphi_{\text{G}}(x,E,d,r_{\text{c}},W)=\phi_{\text{omni}}\left(E,d,r_{\text{c}},W\right)\Phi \left(1.0,E,d,r_{\text{c}}\right)f_{\text{G}}(x,E),$$ (10)

where φ_G_ represents the angular differential fluxes of ground-level muons and *f*_G_ is the ratio between muon fluxes at cos(θ) = *x* and 1.0, which is assumed to be independent of global conditions. According to [46], for extremely high-energy muons, *f*_G_ should be equal to 1/cos(θ), but for lower energies, it should decrease as 1/cos(θ) increases. Fig 13 shows values of *f*_G_ obtained from the measured muon fluxes at *θ* above 78° [43], i.e. 1/cos(*θ*) > 5, divided by the corresponding vertical fluxes calculated by our model. To express this dependence, we propose the following function:

$$f_{\text{G}}(x,E)=\frac{a_{12}\left(E\right)\sqrt{1-\exp\left\{-{\left[\frac{1}{x}\frac{1}{a_{12}\left(E\right)}\right]}^{2}\right\}}-\left\{1-\exp\left[-a_{13}\left(E\right){\left(\frac{1}{x}-1\right)}^{a_{14}\left(E\right)}\right]\right\}}{a_{12}\left(E\right)\sqrt{1-\exp\left\{-{\left[\frac{1}{a_{12}\left(E\right)}\right]}^{2}\right\}}},$$ (11)

where *a*_12_ to *a*_14_ are free parameters depending on energy. The first term of the numerator can be approximated by 1/*x* and *a*_12_ for small and large 1/*x*, respectively, while the second term expresses the decrease of *f*_G_ with increasing 1/*x*. The denominator is introduced to normalize *f*_G_(1,*E*) to 1.0, and *f*_G_ is asymptotic to *a*_12_−1 for large 1/*x*.

The numerical values of *a*_12_ to *a*_14_ were determined from LSq fitting to the data shown in Fig 13 for each energy, and their energy dependences are expressed by Eq (6) for *a*_12_ and the third-order polynomial functions of log_10_(*E*) for *a*_13_ and *a*_14_. The values of *f*_G_ obtained from Eq (11) substituting the calculated *a*_*i*_ are shown in Fig 13. It is evident that Eq (11) can reproduce the experimentally determined values very well.

Substituting the value of *f*_G_ obtained from Eq (11) into Eq (10), the ground-level muon fluxes can be precisely calculated, even for larger zenith angles. However, *f*_G_ can be evaluated only above 11.5 GeV, which is the minimum energy of the muons that were measured in [43]. Below this energy, the ground-level muon spectra are calculated by Eq (1) in the same manner as those in the ideal atmosphere, but their absolute values are normalized to smoothly connect with *φ*_G_ at *E* = 11.5 GeV.

The calculated muon fluxes after considering both high-energy and ground-level corrections are shown in panel (A) of Fig 12. It is evident from the figure that these corrections significantly improve the capability of reproducing experimental data, including the data measured by Jokisch et al. [42], which were not employed in the LSq fitting for determining the *a*_12_ to *a*_14_ parameters. This result indicates the accuracy of our calculation for various conditions, even when experimental data are not available. Note that the ground-level correction is considered when ground instead of ideal atmosphere is selected in PARMA4.0, and, in that case, the integral of the angular differential fluxes with respect to angle is not equal to the corresponding omnidirectional flux.

## Verification of PARMA4.0

### Comparison with EAS Simulation

**Fig 14:**
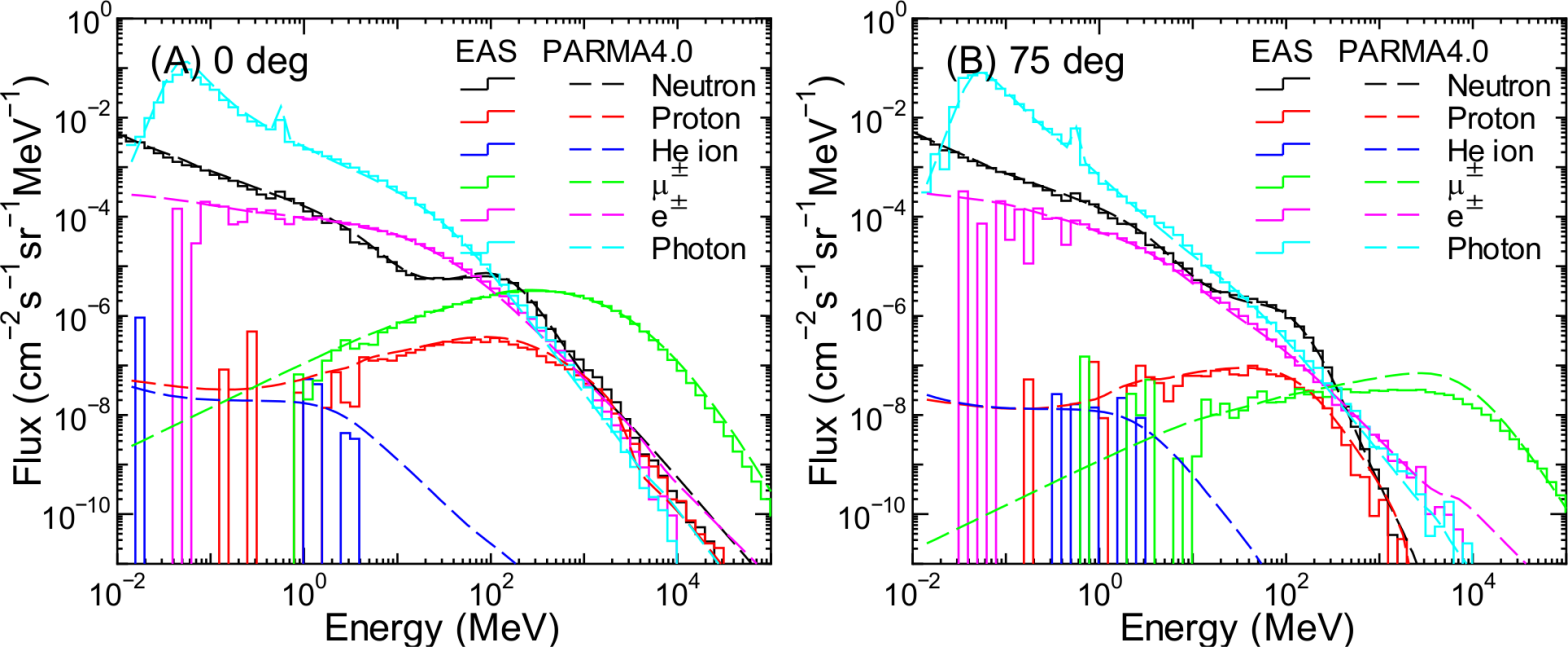
Angular differential energy spectra of cosmic ray fluxes for θ = 0 and 75° at sea level obtained from EAS simulation and PARMA4.0.

**Fig 15:**
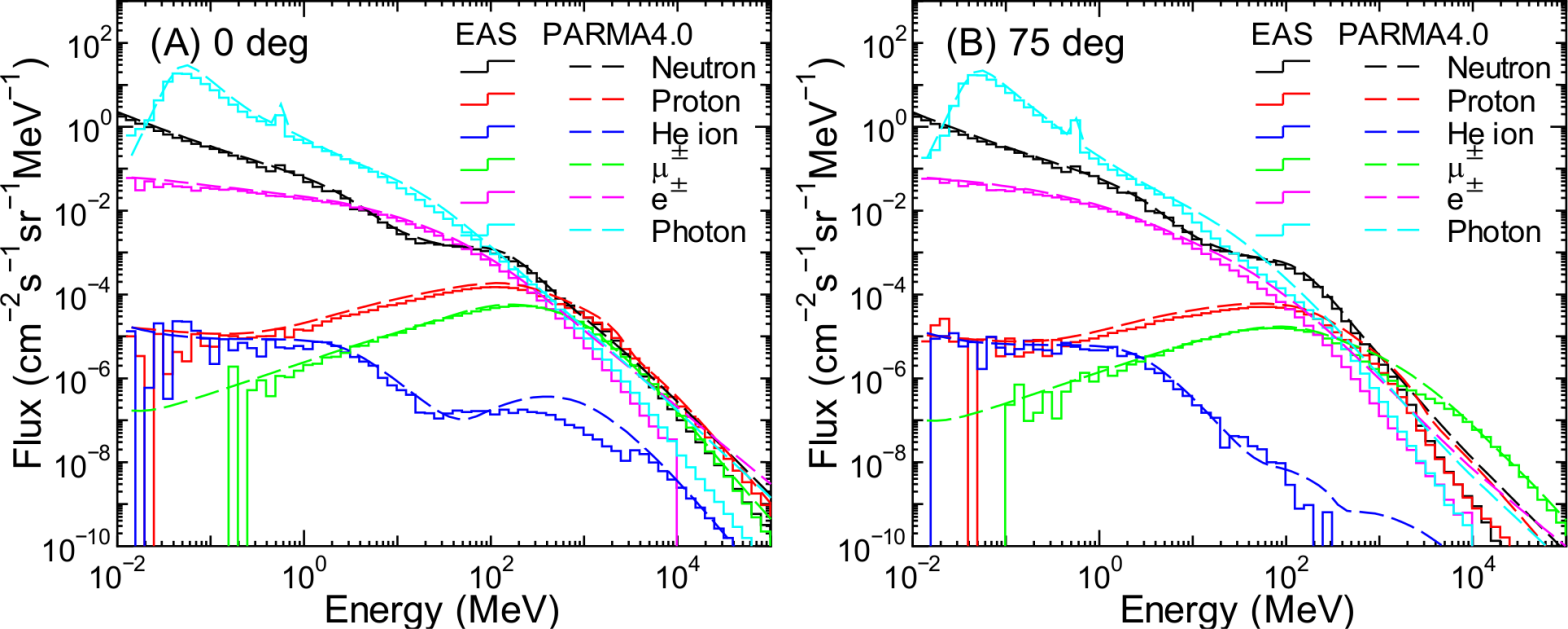
Angular differential energy spectra of cosmic ray fluxes for θ = 0 and 75°at an altitude of 11 km obtained from EAS simulation and PARMA4.0.

**Fig 16:**
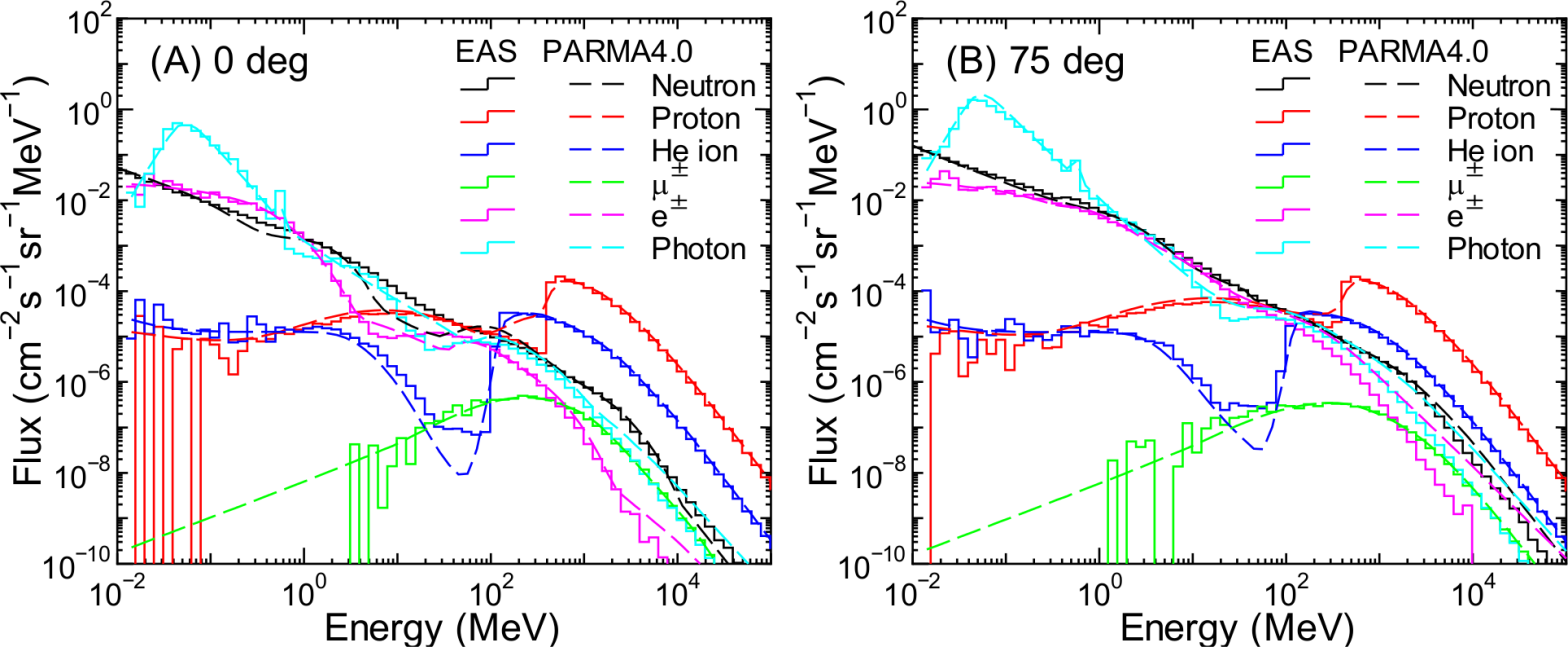
Angular differential energy spectra of cosmic ray fluxes for θ = 0 and 75° at an altitude of 52 km obtained from EAS simulation and PARMA4.0.

Figs 14–16 show the angular differential energy spectra of cosmic ray fluxes for θ = 0 and 75° at the altitudes of 0, 11 and 52 km, respectively, obtained from the EAS simulation and from PARMA4.0. As expected from Figs 2–7, PARMA4.0 can reproduce most EAS data very well and predict smooth curves, even for conditions in which the EAS data are not robust because of large statistical uncertainties. However, some discrepancies are observed in the neutron, e^±^, and photon data over 5 GeV at 0 and 11 km. This discrepancy is attributed to the inaccuracy in PARMA3.0’s calculation of the omnidirectional fluxes in a manner similar to its overestimation of high-energy muon fluxes discussed in the previous section.

To more quantitatively verify the accuracy of PARMA4.0 in reproducing the EAS data, we calculated the coefficients of determination, *R*^2^, of the angular distributions for each particle and global condition using the following equation:

$$R^{2}=1-\frac{\sum_{i,k}{\left[\Phi_{x_{i},E_{k},\text{EAS}}-\Phi \left(x_{i},E_{k}\right)\right]}^{2}}{\sum_{i,k}{\left[\Phi_{x_{i},E_{k},\text{EAS}}-\bar{\Phi_{x_{i},E_{k},\text{EAS}}}\right]}^{2}},$$ (12)

where $\Phi_{x_{i},E_{k},\text{EAS}}$ are the normalized angular distributions obtained from the EAS simulation for the *i*–th angular bin and the *k*-th energy bin, whose central values are *x*_*i*_ and *E*_k_, respectively; $\bar{\Phi_{x_{i},E_{k},\text{EAS}}}$ is the mean value of $\Phi_{x_{i},E_{k},\text{EAS}}$; and Φ(*x*_*i*_,*E*_*k*_) are the corresponding data calculated using Eqs (2) to (5).

**Fig 17:**
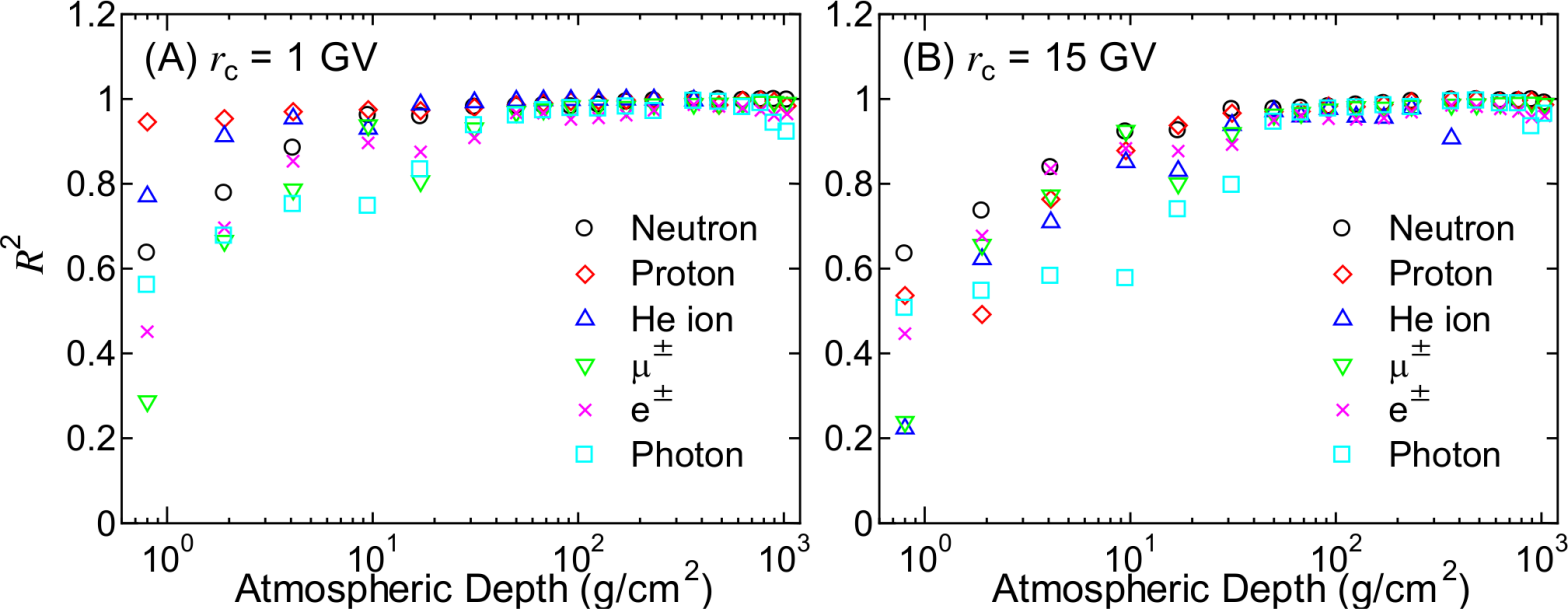
*R*^2^ values calculated by Eq (12) for *r*_c_ = 1 and 15 GV as a function of atmospheric depth.

Fig 17 shows the calculated *R*^2^ for *r*_c_ = 1 and 15 GV as a function of atmospheric depth. Note that the *R*^2^ values for He ions above 364 g/cm^*2*^, i.e., below 8 km, are not plotted because their angular distributions are assumed to be the same as those at 8 km, as previously mentioned. The calculated *R*^2^ values above 50 g/cm^2^, i.e., below 21 km, are very close to 1.0, while they decrease at lower atmospheric depth particularly for the higher *r*_c_ case. Two reasons are considered for this decreasing *R*^2^: the large statistical uncertainties in the EAS data and the difficulty in reproducing the peak structure around the horizontal direction by Eqs (2) to (5). However, primary cosmic rays are the dominant contributor to the fluxes at high altitudes, and PARMA4.0 can reproduce the angular distributions of primary cosmic rays very well, as indicated by the high *R*^2^ values for protons and He ions at *r*_c_ = 1 GV. Based on these considerations, we concluded that PARMA4.0 allows for the instantaneous estimation of the differential cosmic-ray fluxes under most practical situations with an accuracy equivalent to that of the EAS simulation.

### Comparison with Experimental Data

**Fig 18:**
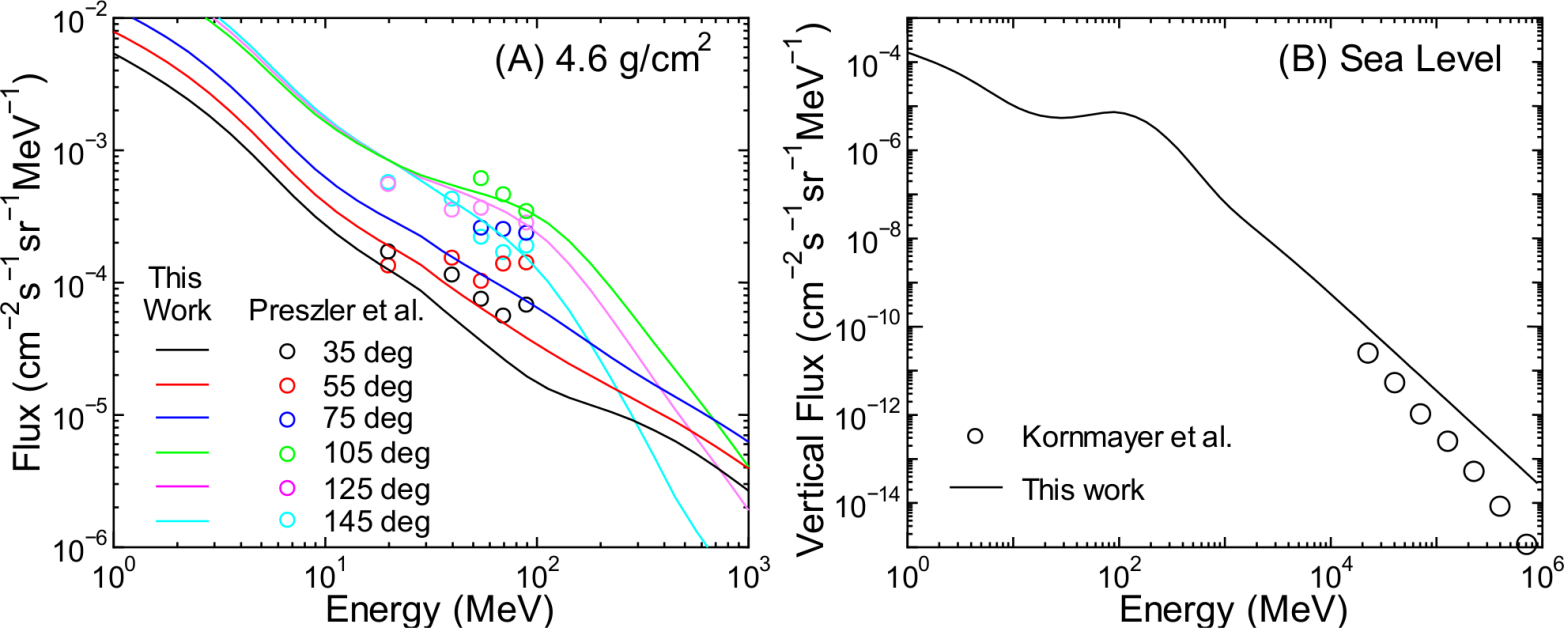
Angular differential energy spectra of neutron fluxes obtained from PARMA4.0 and experiments [47,48]. Data shown in Panel (A) are for various directions, while those in Panel (B) are for the vertical direction.

**Fig 19:**
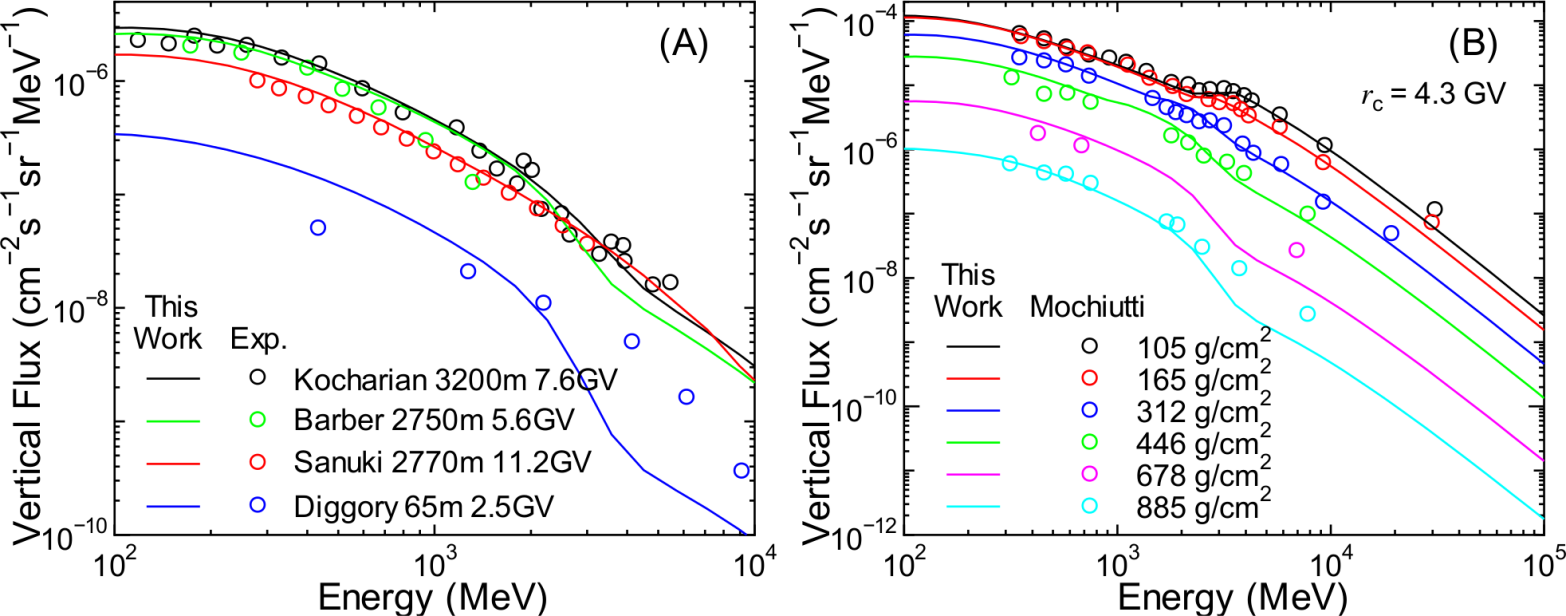
Vertical energy spectra of proton fluxes obtained from PARMA4.0 and experiments [49–53].

**Fig 20:**
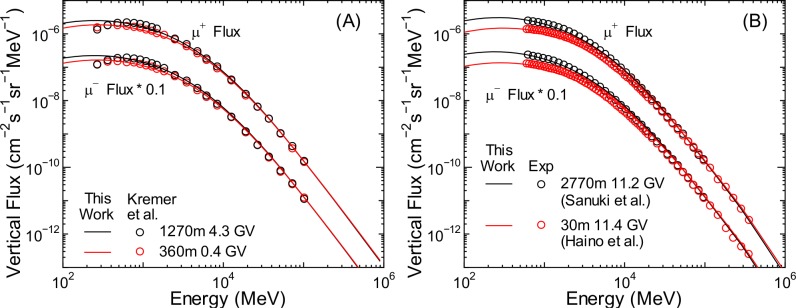
Vertical energy spectra of muon fluxes obtained from PARMA4.0 and experiments [54–56].

**Fig 21:**
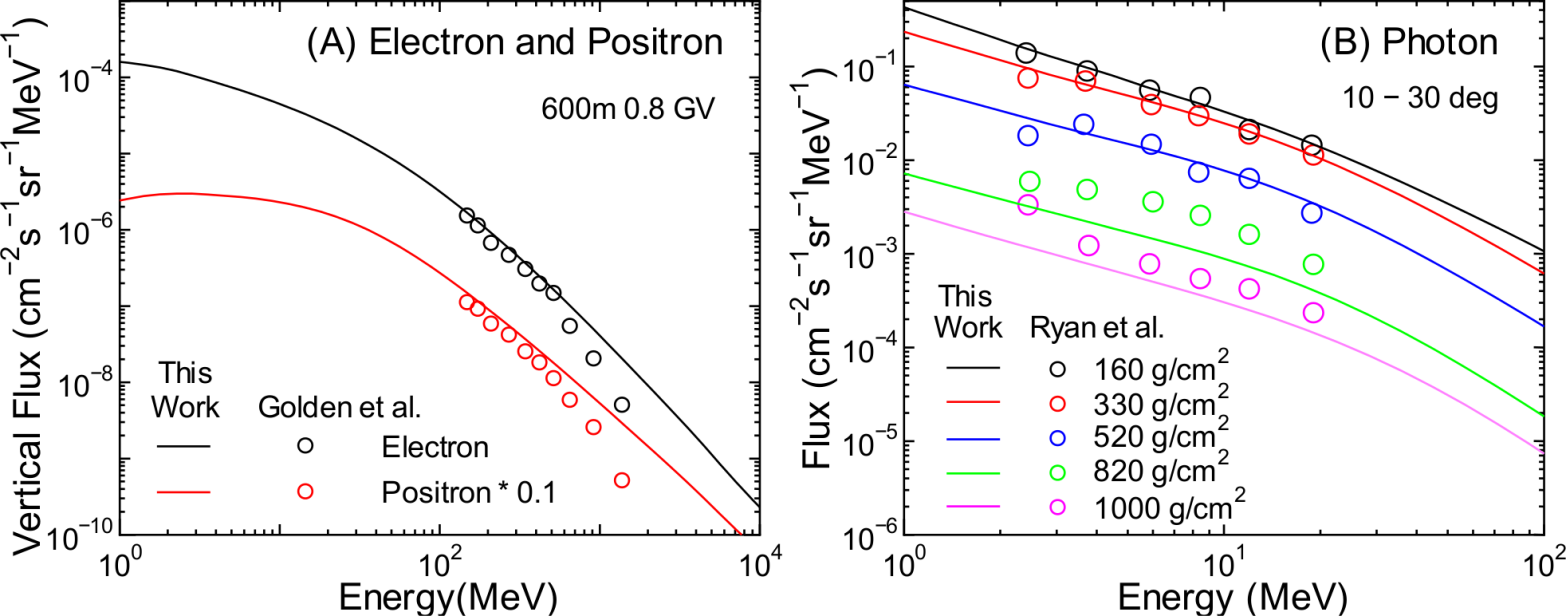
Angular differential energy spectra of electron, positron, and photon fluxes obtained from PARMA4.0 and experiments [57, 58]. Electron and positron fluxes are for the vertical direction, while photon fluxes are for zenith angles between 10° and 30°

Figs 18–21 show a comparison between angular differential energy spectra of cosmic ray fluxes for various conditions obtained from PARMA4.0 and experiments [47–58]. For neutrons, measured angular differential fluxes are available only for very high altitudes or high energies, instead there are many experimental data of omnidirectional neutron fluxes. As shown in panel (A) of Fig 18, the agreements for neutron fluxes at 4.6 g/cm^2^ are generally satisfactory in light of the fluctuation in the experimental data. At such high altitudes, the measured upward fluxes are greater than the downward fluxes, and this trend is well reproduced by the calculation. On the other hand, it is seen from panel (B) of Fig 18 that our calculation significantly overestimates the high-energy vertical neutron fluxes at sea level. This is attributable to the failure of PARMA3.0 in reproducing the omnidirectional neutron fluxes over 5 GeV obtained from the EAS simulation, as discussed previously.

Fig 19 shows that PARMA4.0 can reproduce the measured vertical proton fluxes very well, except for the high-energy data near sea level [50], which cannot be reproduced by EAS simulation neither as shown in our previous paper [17]. The measured spectrum at 105 g/cm^2^ shown in panel (B) has a bump at around 3 GeV owing to the cut-off rigidity, a feature that is closely reproduced by the calculation.

As shown in Fig 20, excellent agreement can be observed between the measurements and calculations for muon fluxes. PARMA4.0 can reproduce detailed features of muon fluxes such as altitude dependence and charge ratio. Considering the agreements observed in muon fluxes for other angles and at higher energies, as shown in Fig 12, we can conclude that PARMA4.0 is well suited for the design of muon radiography.

It is seen from Fig 21 that the results obtained from PARMA4.0 agree with the corresponding experimental data fairly well, although the calculation overestimates and underestimates the high-energy electron fluxes and lower-altitude photon fluxes, respectively. The underestimation of photon fluxes may be owing to the ignorance of the primary cosmic rays above 1 TeV/n in our EAS simulation because such high-energy cosmic rays can generate a substantial number of photons at lower altitudes by inducing extensive air showers, although this hypothesis is rather contradictory to the overestimation of electron fluxes. More experimental data will therefore be necessary to validate the accuracy of PARMA4.0 in calculating electron, positron, and photon fluxes.

## Conclusions

Based on the results of EAS simulation conducted using PHITS, we improved the PARMA model to version 4.0, which facilitates instantaneous estimation of not only omnidirectional but also angular differential energy spectra of cosmic ray fluxes anywhere in Earth’s atmosphere at nearly any time. Both the presence of realistic ground and of the virtual black hole can be considered in the calculation of ground-level fluxes. The angular distributions of ground-level muons at larger zenith angles are specially determined using an optional function developed on the basis of experimental data. The accuracy of PARMA4.0 was carefully examined using multiple sets of experimental data obtained under various global conditions. The agreements between the calculated and measured data are generally satisfactory, particularly for muons, although some disagreements are observed in high-energy fluxes.

This extension of PARMA enlarges its applicability to wider research areas, such as the design of cosmic-ray detectors, muon radiography, soil moisture monitoring, and cosmic-ray shielding calculations. For example, it has already been used in the calculation of the attenuation length of underground muons [59]. PARMA4.0 is available freely and is easy to use, as implemented in the new version of EXPACS.
